# Analysis of patient preferences on patient–provider interactions through the OpenNotes online portal in dermatology

**DOI:** 10.1016/j.ijwd.2021.10.001

**Published:** 2021-10-05

**Authors:** Christopher Henderson, Zachary P. Nahmias, Alan Fossa, Ethan Barnes, Susan Huang

**Affiliations:** a Harvard Medical School, Boston, Massachusetts; b Dermatology Department, Samaritan Medical Center, Watertown, New York; c Post-Baccalaureate Office, Thomas Jefferson University, Philadelphia, Pennsylvania

**Keywords:** OpenNotes, dermatology, patient note, privacy, survey

## Abstract

*Background:* Many medical centers are beginning to use OpenNotes (ON) to empower patients. However, there is a lack of literature reviewing the ON system in dermatology and any differences in attitudes between men and women. If so, it is uncertain what concerns are more important to female patients. Given the complex lexicon of notes in dermatology, the outpatient setting of dermatology practices, and the often-complex nature of treatment regimens, investigation was merited.

*Objective:* This paper aimed to evaluate a survey of dermatologic patients on their attitudes toward the ON system.

*Methods:* From July through October 2015, 333 dermatologic patients at the Beth Israel Deaconess Medical Center completed an anonymous, voluntary, 25-question survey of the ON system while in the waiting room. Approximately 60% of respondents were female and 40% were male. Respondents were older, with 27% age >65 years, 21% between 56 and 65 years, 16% between 46 and 55 years, 17% between 36 and 45 years, 14% between 26 and 35 years, and 4% between 18 and 25 years. Eighty-five percent of respondents were white, and 73% had, at minimum, graduated from college.

*Results:* Patient response to ON was positive, with 93% agreeing ON is a good idea. Of the patients who accessed their own notes (69% of respondents), 99.6% desired continued access. In addition, 85.6% of patients felt ON allowed them to control their own health, and 70% reported increased confidence in their dermatologist. Nineteen percent of respondents thought ON presented a privacy concern.

*Conclusion:* The results showed that female patients strongly desire access to their medical records, but concerns about privacy and security exist. Preliminary analysis by a statistician did not find any statistically significant variations between men and women within the results of the survey. Due to the wide agreement in responses, it is unlikely that there are significant differences in opinion on ON between men and women.

**What is known about this subject regarding women and their families:**

• OpenNotes is an online portal that allows patients access to their medical records.
• Initial trials showed a generally favorable response to OpenNotes’ implementation.
• There has been an increased demand for increased patient access to medical records in recent years.

**What is new from this article as messages for women and their families?**

• We did not find any statistically significant variations between men and women regarding attitudes toward OpenNotes (ON) within dermatology.
• Dermatology patients overwhelmingly prefer continued access to ON, with few patients reporting major concerns.
• Patients report that access to their medical records through ON allows them better understanding of their health and increases their perceived control over their own health care.

Alt-text: Unlabelled box

## Introduction

In 2010, the Beth Israel Deaconess Medical Center (BIDMC) launched OpenNotes (ON), an online tool that allows patients to directly access their physicians’ notes in the primary care setting, with the aim of increasing transparency between patients and providers. As a web-based portal, ON allows patients to directly access their outpatient medical notes and can contribute to patients’ treatment (Delbaco et al., 2010). Patients gained the right to access their health records after federal passage of the Health Insurance Portability and Accountability Act of 1996 and the 21^st^ Century Cures Act of 2016. Additionally, the American Recovery and Reinvestment Act requires health care providers to provide patients with timely electronic access, within 4 business days of its availability to the eligible professional, to their health information, including laboratory test results, problem lists, medication lists, and allergies (Murphy-Abdouch, 2015). However, studies have shown that only 0.4% to 2% of patients spontaneously request access to their records, although at least 75% are interested in easier access (Ross and Lin, 2003). In addition, the incipient stage of this regulation produced several questions regarding cost for access to records online (Murphy-Abdouch, 2015), including how much health care providers can charge for access and whether charges are a one-time bulk payment or a per-page cost.

Initial study results after the 2010 ON trial in the primary care setting showed that of 13,564 patients surveyed, most accessed at least one note (84% at BIDMC, 82% at Geisinger Health System in Pennsylvania, and 47% at Harborview Medical Center in Washington). In addition, 77% to 87% of patients reported feeling more in control of their care, and 60% to 78% reported increased medication adherence (Delbanco et al., 2012). Of the 105 physicians surveyed in this trial, few reported longer visits or required more time to complete notes (Delbanco et al., 2012). Although this study showed that privacy concerns by patients exist, these concerns are limited and do not seem to affect access (Vodicka et al., 2013).

Following these generally positive results, a fully accessible notes policy was adopted by institutions such as the Mayo Clinic, Cleveland Clinic, Dartmouth, Kaiser Permanente Northwest, and MD Anderson Cancer Hospital (Walker et al., 2015) and has spread beyond adult primary care to new specialties, such as dermatology and pediatrics (Sarabu et al., 2018). Furthermore, a survey of psychotherapy patients’ experience with open therapy notes has also shown promising results (O'Neill et al., 2019).

A 2016 study by the authors of the current study found positive results with a few minor concerns (Henderson et al., 2016), and a more recent survey of dermatopathologists found mixed results (Shucard et al., 2020). However, evaluations of ON in the field of dermatology overall remain sparse. Given the complex lexicon of notes in dermatology, the outpatient setting of most dermatology practices, the longitudinal component of skin health, and the often-complex nature of treatment regimens, the BIDMC dermatology patient experience with ON merited investigation (Lott et al., 2015; Miller, 2020). Individuals with access to their health information are better able to maintain prescribed treatments, control chronic health problems, correct errors in their health records (Bell et al., 2020; Bourgeois et al., 2019), track progression in their treatment plan, and personally contribute their records to research (DelBanco et al., 2010).

Dermatology, with its generally large panel size, may also have benefits for physician workload by allowing patients to take some responsibility over their own care, thereby improving patient outcomes (Murray et al., 2007; Dorrell et al., 2019). However, studies of patient-accessible medical records suggest modest improvements in doctor–patient communication, adherence, patient empowerment, and patient education, with patients finding parts of their medical records confusing. Ross and Lin (2003) found that few medical patients thought the experience was confusing or upsetting, but a significant portion of psychiatric patients became more worried and pessimistic after reading their records.

There is also a well-documented history of medical providers downplaying female patients’ reports of pain intensity (Chen et al., 2008; Hoffmann and Tarzian, 2001). Providers may harbor similar gendered views on access to medical records. We evaluated the patient experience with ON in the ambulatory dermatology setting to determine the extent of usage, the reasons for ON access by patients, the change in patient perception of their dermatologist, how ON affected patients’ perception of their health, and whether patient behaviors are influenced by ON access.

All dermatology patients at the BIDMC obtained access to their dermatologists’ clinical notes starting January 2014. All 17 dermatologists in the department participated in the program. The median and mode for outpatient medical visit length is approximately 15 minutes. We hypothesized that the ON system could be a way to enhance patients’ education and understanding of their care and dermatologists’ recommendations. With more effective education, adherence to treatment regimens could plausibly increase. For instance, one prior study showed that a significant proportion of patients apply their topical steroid medications incorrectly; 36.4% of patients applied the medication too frequently, 37.7% of patients changed the site of application over the course of treatment, and no patients knew to use the fingertip unit method to assess dose (Jeziorkowska et al., 2015).

Our study can encourage the use of ON in the dermatology clinic. Patient survey comments can assuage provider concerns on whether ON allowed patients to be better informed about their diagnosis, care plan, and follow-up. Additionally, this study can show the impact that the transparency and empowerment of ON can have on patient confidence. Given the federal mandate for electronic health records, these data can reassure dermatology clinics that medical note transparency is a net boon for the patient–physician relationship.

## Methods

Surveys were collected from July through October 2015 in the Department of Dermatology at BIDMC after institutional review board approval was obtained. After arriving for outpatient appointments, patients established with the clinic since January 2014 (the time of rollout of ON at the BIDMC dermatology department) were asked by dermatology research staff to complete a voluntary, anonymous survey before their visit. Patients filled out the survey in the waiting room; these were then returned to a locked collection bin. Survey subjects were established dermatology patients since January 2014 and would have had at least one visible dermatology note through ON.

The survey was adapted from the original 2010 ON survey and consisted of 25 questions, most of which were Likert-type questions. In all, our survey included “check all that apply” questions, open-ended response questions, yes/no questions, and Likert-type scale questions. In total, 333 surveys were collected, with 10 surveys not containing a response to any question. The data were identified by a unique survey ID, and analysis was performed using SAS software, version 9.3 (SAS Institute Inc., Cary, NC).

## Results

**Figure 1:**
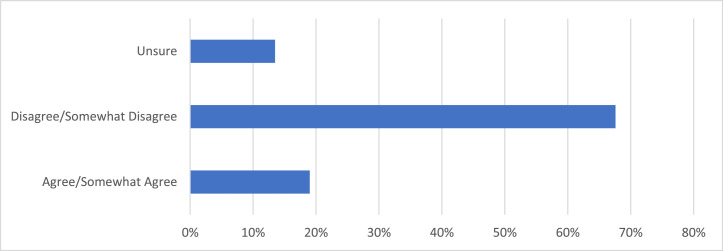
**Patient Response to the Survey Question “I am concerned about the privacy of my visit notes on OpenNotes?”** 333 dermatology patients at the Beth Israel Deaconess Medical Center were surveyed over potential privacy concerns regarding their medical notes. 67.6% of respondents disagreed that OpenNotes access presented a privacy concern, 19.0% agreed that it presented a privacy concern, and 13.5% were unsure.

**Figure 2:**
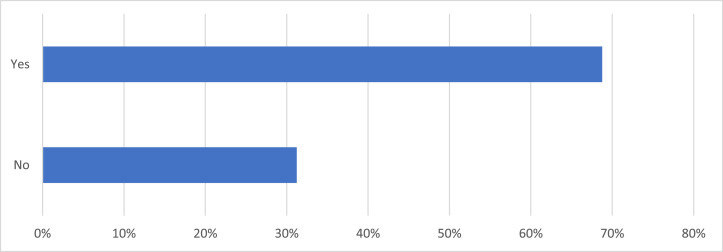
**Patient Response to the Survey Question “Did you look at any of your dermatologist's visit note(s) on PatientSite?”** 333 dermatology patients at the Beth Israel Deaconess Medical Centers were surveyed on whether they had previously accessed the OpenNotes online application, PatientSite. 68.8% of respondents claimed that they had previously accessed PatientSite, while 31.2% of respondents reported that they had not.

Of 323 responses, 92.9% agreed that online note availability was a good idea, and a further 4.0% somewhat agreed. More respondents were unsure (2.2%) than disagreed (0.3%) or somewhat disagreed (0.6%) with ON access for patients. When queried regarding the impact on visit privacy, 67.6% disagreed that ON access presented a privacy concern, whereas 19.0% agreed and 13.5% were unsure (Fig. 1). Patients were asked whether they had previously accessed the ON online application, ON PatientSite. ON PatientSite is an online application that allows patients to manage their health care virtually. Approximately two-thirds of patients stated they had previously accessed the online application, and within this group of patients, 99.6% expressed desire for continued access (Fig. 2). Patients believed that their notes always (66.8%) or usually (26.6%) described the clinic visit accurately, with only 0.4% of patients having expressed the notes never accurately described their visit, 2.2% believed the notes were sometimes accurate, and 4.0% were unsure.

Patient perceptions of note quality were strongly positive, with 70.6% reporting that notes were easy to understand and another 25.9% reporting that notes were somewhat easy to understand. Patients largely agreed (86.9%) or somewhat agreed (11.8%) that access to their dermatologist's notes allowed them to better understand their health and medical conditions. This seemed to translate to patient perceptions of health control: 85.6% agreed and 12.2% somewhat agreed that dermatology note access allowed them to feel more in control of their health.

**Figure 3:**
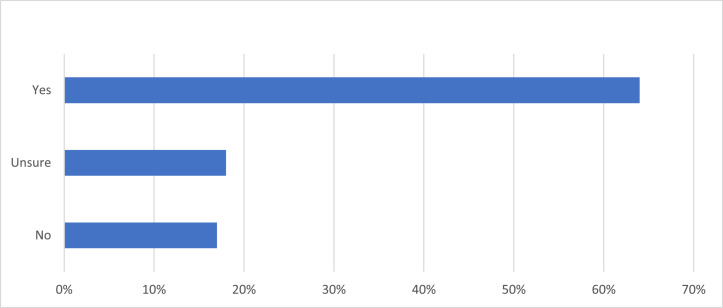
**Patient Response to the Survey Question “Did accessing the note(s) lead you to take better care of your skin?**” 333 dermatology patients at the Beth Israel Deaconess Medical Centers were surveyed on whether they believed that note access allowed them to take better care of their skin. 64.2% of respondents reported that it allowed them to take better care of their skin, 17.2% reported that it did not allow them to take better care of their skin, and 18.1% were unsure.

**Figure 4:**
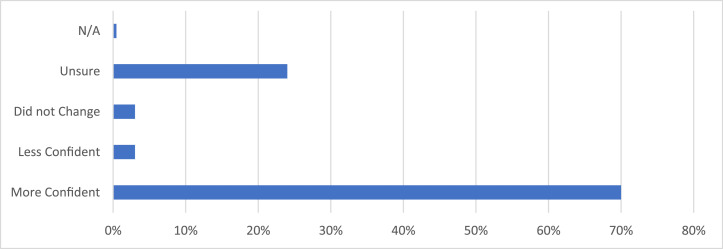
**Patient Response to the Survey Question “Did reading the dermatologist's note change your confidence level in your dermatologist?”** 333 dermatology patients at the Beth Israel Deaconess Medical Centers were surveyed on whether reading their patient note changed their confidence in their dermatologist. 69.7% claimed it made them more confident in their dermatologist, 2.75% reported that it did not change their opinion, 2.75% claimed that it made them less confident in their dermatologist, and 24.3% were unsure.

Similarly, 64.2% agreed that note access allowed them to take better care of their skin (Fig. 3). When asked about notes provoking anxiety, only 2.7% agreed and 4.5% somewhat agreed. Note clarity seemed high as well: Only 0.9% agreed and 3.2% somewhat agreed that dermatology notes caused them to feel confused, 85.1% disagreed that notes were confusing, and 9.5% somewhat disagreed. When asked if they contacted their dermatologist's office secondary to reading their notes, only 5.4% said yes. When asked if reading their provider's notes changed confidence in their dermatologist, 69.7% reported that the notes caused them to be more confident in their dermatologist, 2.8% reported less confidence, 2.8% reported that their confidence did not change, and 24.3% were unsure (Fig. 4).

The reasons for reading dermatologists’ notes varied among respondents. Our survey provided nine reasons, of which all, some, or none could be selected by respondents. Of the respondents, 56.4% reported that they were curious, 30.4% wanted to remember the visit, 49.3% wanted to know about their health, 49.3% wanted to be sure they understood their diagnosis, 44.1% wanted to understand their treatment plan, 42.7% wanted to know what their doctor was thinking, and 2.6% reported no particular reason. There were minimal responses to the reason “I have a right to see what's in my medical record.” For the few patients who did give a unique response, none were significant enough to be included in the results (e.g., the patient enjoyed learning new scientific terms).

Our survey also included an opportunity for patients to leave open-ended feedback. One query asked, “did something happen (good or bad) as a result of reading your notes?” There was also a query for additional information. Several patients wrote that accessing their dermatology notes allowed them to understand their condition or follow-up care better. Reading the dermatologists’ notes allowed several respondents to remember to make their follow-up appointment. Several wrote that they felt more informed or were reassured by the notes. A few patients commented that they found errors in the notes. We also asked the one-third of patients who chose not to read their dermatologist's notes why they did not. Only 1.0% reported this was due to a lack of Internet access, with 99% reporting this was not a reason. In addition, 3.9% reported that they did not believe the notes would be useful, 13.6% forgot their notes were available, 1.9% could not find the online portal, 7.8% were too busy, 1% thought the notes would cause anxiety, and 18.5% had no reason.

Of the 295 respondents who reported gender, 60.0% identified as female and 40.0% identified as male. Preliminary analysis by a statistician did not find any statistically significant variations between men and women within the results of the survey. Female patients did report that there were some concerns about privacy; however, most responses did not show such a concern. Moreover, a negative response about privacy did not predicate a negative attitude toward ON or a decreased desire for continued access. Because responses to several questions were nearly unanimous, it is unlikely that there are any significant differences in attitudes and concerns regarding ON between men and women. The most common age range was >65 years (27.8% of respondents); 21.3% were age 56 to 65 years, 16.2% were 46 to 55 years, 16.8% were 36 to 45 years, 14.1% were 26 to 35 years, and 3.8% were 18 to 25 years.

Patients were generally more educated than the general population, with 46.4% reporting having at least some graduate school, 25.9% having finished a 4-year college, and 21.5% having finished some college. Most patients (86.3%) considered themselves white, 6.3% considered themselves black, and 3.9% considered themselves other. As a separate question, 96.2% did not consider themselves of Spanish/Hispanic/Latino ethnicity, whereas 3.8% of patients did (Table 1).

**Table 1:** Demographics of survey respondents.

	Overall, n (%) (n = 333)	Patients who read at least one dermatology note, n (%) (n = 229)	Patients who read no dermatology notes, n (%) (n = 104)
Demographics*			
*Overall skin health*			
Excellent	7 (2.5)	4 (2.2)	3 (3.1)
Very good	47 (16.8)	32 (17.6)	15 (15.3)
Good	149 (53.2)	91 (50)	58 (59.2)
Fair	63 (22.5)	45 (24.7)	18 (18.4)
Poor	13 (4.6)	9 (4.9)	4 (4.1)
*Education*			
College graduate	73 (26.1)	47 (25.8)	26 (26.5)
Some graduate level education	132 (47.1)	81 (44.5)	51 (52)
High school graduate	74 (26.4)	53 (29.1)	21 (21.4)
Less than high school	1 (0.4)	1 (0.5)	0 (0)
*Age, years*			
18–25	11 (3.9)	9 (4.9)	2 (2)
26–35	39 (13.9)	27 (14.8)	12 (!2.2)
36–45	48 (17.1)	28 (15.4)	20 (20.4)
46–55	46 (16.4)	25 (13.7)	21 (21.4)
56–65	60 (21.4)	42 (23.1)	18 (18.4)
65+	76 (27.1)	51 (28)	25 (25.5)
*Sex*			
Female	167 (59.6)	109 (59.9)	58 (59.2)
Male	113 (40.4)	73 (40.1)	40 (40.8)
*Race/ethnicity*			
Asian	4 (1.4)	3 (1.6)	1 (1)
Black	13 (4.6)	9 (4.9)	4 (4.1)
Hispanic or Latino	10 (3.6)	8 (4.4)	4 (4.1)
White	239 (85.4)	153 (84.1)	86 (87.8)
Other/multiple races	14 (5)	9 (4.9)	5 (5.1)

⁎ Fifty-three patients with missing demographics information were excluded.

## Discussion

Similar to the 2010 study conducted among primary care providers, our results show that 93% of respondents agreed that ON access is a good idea, which demonstrates that patients have an overall favorable outlook on the ON platform. Almost all patients wanted continued access, and providing ON access may be an avenue to increase patient satisfaction. Although there were initial fears from providers about potential negative consequences to the physician–patient relationship from ON, this was not the case as viewed by our surveyed patients. More than 90% of patients in our study believed their notes to be always or usually accurate, and >95% believed their notes were easy or somewhat easy to understand.

Almost 70% of patients felt more confident in their dermatologist because of ON access, with <3% feeling less confident. Preliminary analysis showed that there was no difference in attitudes between men and women. There may have been concerns that allowing open access to medical records would uncover possible gendered attitudes by providers on this issue, similar to the issue of discrimination on the basis of sex in pain management. However, the results should help to assuage any similar fears on the use of ON. Metrics such as improved confidence in their provider should dampen fears of erosion of the physician–patient relationship.

Access to medical records is an avenue through which patients can increase knowledge of their own health status and improve their autonomy. Almost all patients felt more in control of their health as a result of reviewing their records online, and >90% believed they were better able to care for their skin health as a result of such electronic access. Although an individual dermatology visit is one discrete point in time, the patient's care of her or his health condition continues after the visit. Access to ON helps with this continuum of care and serves as a resource for the patient. Patients can access ON as long as they have Internet access and can review their note as many times as needed. ON can serve as a reference for the prescribed treatment regimen and counsel and improve patients’ medical literacy regarding their diagnosis.

Privacy was reported as a concern among approximately one-third of patients. Ensuring security will be important as electronic access expands in our health care system. Practices will need to maintain an open and consistent dialogue with patients about the steps used to protect patient privacy. Cybersecurity consultation services will likely increase in demand as electronic health records become universal in the United States and the threat of cyberattacks increases concordantly.

There were several limitations to the present study. The paper was not formally analyzed and extrapolated on a gender basis, thus limiting its generalizability. The inclusion of only one ambulatory center in an urban academic practice may not be generalizable to other practice settings. Most respondents were white and highly educated relative to the general U.S. population, and nearly half were age ≥56 years. A low sample size among less-educated patients, as well as among patients not identifying as white, might affect the generalizability of our study. Our sample size was not large enough to make inferences on attitudes on ON among women with a high degree of confidence. Furthermore, the approach used in our study relied on patient self-reported data.

## Conclusion

Several concerns are left unanswered by this preliminary analysis. Although this study did not find any significant differences between men and women regarding ON, variations may exist in both why and how men and women choose to engage with online medical records. Additional research is needed on this topic. Moreover, although patients prefer open access to their records and believe it to be beneficial, a causal relationship has yet to be established. Highly educated patients who had previously read their clinical notes were unable to correctly select their diagnosis when tested, despite previously reporting a feeling of confidence and understanding of the note (Yanovsky et al., 2020). Additional literature should be published on whether access to notes truly improves patient outcomes and assists the physician–patient relationship.

Not all ethical concerns regarding patient privacy have been fully addressed; allowing family to gain automatic access to patient notes may lead to patients withholding information out of fear of discovery (Bourgeois et al., 2018). This problem may worsen in the coming years as proxy access for nonpatients becomes more widespread (Desroches et al., 2020).

Most literature on ON has studied patients at the same three pilot sites located in Boston (BIDMC), Seattle (Harborview Medical Center), and central Pennsylvania (Geisinger Health System). As a result, several of the largest studies have skewed toward white patients and are overwhelmingly focused on well-educated patients. The groups underrepresented by these surveys (nonwhite and less educated) are those who may stand to gain the most from accessing their notes (Bell et al., 2016; Blease et al., 2020; Walker et al., 2019). Since its launch, >90 health care organizations have adopted ON, and this trend is likely to continue to evolve (Bourgeois et al., 2018; Kriegel et al., 2020). Additional efforts are needed to study these locations to gain a more representative view of the country.

## Conflicts of interest

During the time at which this study was conducted and the time the data were analyzed for the study, author Alan Fossa was employed by the OpenNotes research group within the Beth Israel Deaconess Medical Center. The OpenNotes research group (opennotes.org) openly advocates for the sharing of clinical notes with patients, which is the subject under question in this study. The author's assistance with this study was a professional favor and not part of his normal work requirements, and he is no longer employed by the OpenNotes research group or Beth Israel Deaconess Medical Center.

## Funding

None.

## Study approval

The author(s) confirm that any aspect of the work covered in this manuscript that has involved human patients has been conducted with the ethical approval of all relevant bodies.

## Appendix Supplementary materials

Image, application 1
