# Supplementary material for: Analysis of patient preferences on patient–provider interactions through the OpenNotes online portal in dermatology
Source: Int J Womens Dermatol. 2021 Oct 5;7(5Part B):793–8. doi: 10.1016/j.ijwd.2021.10.001 (PMC8714571; doi:10.1016/j.ijwd.2021.10.001)
Supplement: Supplementary file 1 [file mmc1.docx]

Analysis of patient-provider interaction through the OpenNotes online portal in dermatology

Archive of Dermatological Research

Christopher Henderson MD MPhil,^1^ Zachary P. Nahmias MD,^2^ Alan Fossa MPH,^1^ Ethan Barnes, BA,^3^ and Susan Huang MD^1^

^1^ Harvard Medical School

^2^ Samaritan Medical Center, Watertown, NY

^3^ Thomas Jefferson University

**Correspondence:**

Ethan L. Barnes

Thomas Jefferson University

[ethan.barnes@jefferson.edu](mailto:znahmias@shsny.com)

BIDMC Dermatology OpenNotes Survey for Patients

For patients seen by the BIDMC Department of Dermatology

1. In general, making visit notes available to patients on PatientSite is a good idea.

Disagree Somewhat disagree Somewhat agree Agree Don’t know

Participating patients only

2. Did you look at any of your dermatology doctor’s visit note(s) on PatientSite?

Yes 🡪 Q5 No 🡪 Q3 🡪 Q4 🡪 Q

I did not have any notes to look at because I did not see my doctor since notes were made available (date notes from dermatology were made available in OpenNotes?). 🡪

3. What was the reason you did not look at your dermatology notes? (check all that apply)

I didn’t think it would be useful

I forgot my notes were available

I tried, but I could not find my notes on PatientSite

I was too busy

I thought reading the notes would make me nervous or anxious

Another reason

No particular reason

Another reason proceed to 🡪 Q4

4. Please tell us more about why you didn’t read your doctor’s notes.

All Responses - blank or empty proceed to 🡪 Q

5. Why did you read your visit notes? (check all that apply)

I was curious

I wanted to remember what happened in the visit

I wanted to know about my health

I have a right to see what’s in my medical record

I wanted to check the notes to see if they were right

I wanted to be sure I understood what the doctor said

I wanted to know what my doctor was thinking

No particular reason

Other reason (please explain) __________

6. I would like to continue to be able to see my dermatologist’s notes online.

Yes No

7. How often did the note(s) accurately describe the visit?

Never Sometimes Usually Always Don't know

8. How easy was it to understand your dermatologist’s notes?

Very difficult Somewhat difficult Somewhat easy Very easy Don’t know

9. I understand my health and medical conditions better.

Disagree Somewhat disagree Somewhat agree Agree Don’t know

10. I remember the plan for my skin care better and feel more in control of my skin health.

Disagree Somewhat disagree Somewhat agree Agree Don’t know

11. I take better care of my skin and like the results better as a result of having access to my visit notes with dermatology.

Disagree Somewhat disagree Somewhat agree Agree Don’t know

12. Did reading the dermatologist’s note change the way you feel about your doctor?

I feel much worse I feel somewhat worse I don’t feel better or worse I feel somewhat better I feel much better

13. Did something happen (good or bad) as a result of reading your notes? Please describe your experience here:

14. In general, how would you rate your overall health?

Excellent Very good Good Fair Poor

15. How would you rate your overall skin health?

Excellent Very good Good Fair Poor

16. What is the highest grade or level of school that you have completed?

8th grade or less Some high school, but did not graduate High school graduate or GED Some college or 2-year degree 4-year college graduate Some graduate school Masters or Doctoral degree

17. Are you of Spanish/Hispanic/Latino ethnicity?

Yes No

18. What do you consider to be your racial background? (check all that apply)

White Black or African American American Indian or Alaskan Native Asian Native Hawaiian or Pacific Islander Other

19. If there is anything else that you would like us to know about you, or other comments you would like to make, please write them here:

Thank you for participating in this survey and helping us better serve you.
